# Participatory Workplace Interventions Can Reduce Sedentary Time for Office Workers—A Randomised Controlled Trial

**DOI:** 10.1371/journal.pone.0078957

**Published:** 2013-11-12

**Authors:** Sharon Parry, Leon Straker, Nicholas D. Gilson, Anne J. Smith

**Affiliations:** 1 School of Physiotherapy, Curtin University, Perth Western Australia, Australia; 2 School of Human Movement Studies, University of Queensland, Brisbane Queensland, Australia; University of Bath, United Kingdom

## Abstract

**Background:**

Occupational sedentary behaviour is an important contributor to overall sedentary risk. There is limited evidence for effective workplace interventions to reduce occupational sedentary time and increase light activity during work hours. The purpose of the study was to determine if participatory workplace interventions could reduce total sedentary time, sustained sedentary time (bouts >30 minutes), increase the frequency of breaks in sedentary time and promote light intensity activity and moderate/vigorous activity (MVPA) during work hours.

**Methods:**

A randomised controlled trial (ANZCTR number: ACTN12612000743864) was conducted using clerical, call centre and data processing workers (n = 62, aged 25–59 years) in 3 large government organisations in Perth, Australia. Three groups developed interventions with a participatory approach: ‘Active office’ (n = 19), ‘Active Workstation’ and promotion of incidental office activity; ‘Traditional physical activity’ (n = 14), pedometer challenge to increase activity between productive work time and ‘Office ergonomics’ (n = 29), computer workstation design and breaking up computer tasks. Accelerometer (ActiGraph GT3X, 7 days) determined sedentary time, sustained sedentary time, breaks in sedentary time, light intensity activity and MVPA on work days and during work hours were measured before and following a 12 week intervention period.

**Results:**

For all participants there was a significant reduction in sedentary time on work days (−1.6%, p = 0.006) and during work hours (−1.7%, p = 0.014) and a significant increase in number of breaks/sedentary hour on work days (0.64, p = 0.005) and during work hours (0.72, p = 0.015); there was a concurrent significant increase in light activity during work hours (1.5%, p = 0.012) and MVPA on work days (0.6%, p = 0.012).

**Conclusions:**

This study explored novel ways to modify work practices to reduce occupational sedentary behaviour. Participatory workplace interventions can reduce sedentary time, increase the frequency of breaks and improve light activity and MVPA of office workers by using a variety of interventions.

**Trial Registration:**

Australian New Zealand Clinical Trials Registry ACTN12612000743864.

## Introduction

There is a growing understanding that high levels of total sedentary time and sustained sedentary time (or lack of breaks in sedentary time) and low levels of light intensity physical activity are associated with poor health independent of moderate/vigorous activity [1]–[6]. Epidemiological studies have found increased cardiometabolic risk factors with increased overall sedentary time, fewer breaks and reduced light activity [2], [7]–[9]. Recent laboratory studies have found that interrupting sustained sedentary time with short bouts of treadmill walking resulted in improved glucose metabolism in overweight individuals [10] and increased energy expenditure in normal weight individuals [11], suggesting that relatively small changes in activity level and pattern have the potential to modify adverse health risks.

Exposure to sedentary behaviours (awake activities such as sitting which expend less than or equal to 1.5 METS [12]) is thought to have increased in modern times due to changes in land use, leisure activities, active transport, technological advancements and the workforce proportion in sedentary occupations [13]–[15]. Indeed occupational sedentary exposure is being recognised as an important risk factor [16]–[21].

The workplace has been used to conveniently implement health promotion interventions [22]. Workplace interventions have successfully addressed work risks associated with manual handling tasks [23] and computing tasks [24], typically aimed at reducing musculoskeletal symptoms, injuries and absenteeism [25], [26]. Workplace interventions have also successfully addressed risks associated with alcohol, smoking and nutrition [27]–[30] as well as the promotion of moderate/vigorous physical activity (MVPA) [31] supporting suggestions that the workplace may be a suitable site to implement programmes to reduce sedentary behaviours [32].

The recognition of the importance of sedentary time, and the success of workplace interventions for other health issues, has highlighted the need to develop workplace interventions that aim to reduce sedentary time, increase breaks in sedentary time and incorporate light physical activity [5], [33], [34]. In a 2010 review of the intervention studies to reduce sitting time at work, it was found that there were very few quality intervention studies, with no intervention demonstrating a significant reduction in sitting time [33]. One potential reason for the lack of evidence of success was that sitting time was mainly self-reported [33]. Objectively measured sedentary time [35] and pattern of exposure [36], [37] may provide more robust evidence. Indeed, recent studies to reduce workplace sitting time by use of standing desks [38] and break-prompting software [39], using objectively rather than self-report measures have found reduced sitting time and improved frequency of breaks in sedentary time.

There have been three main approaches to improving workplace physical activity and sedentary behaviour. The first approach has traditionally aimed to incorporate MVPA into the working day during transport to and from work and during lunch and other breaks between productive work time [40]–[42]. For example, a recent study examined the effect of a workplace pedometer challenge [31]. The second traditional approach has been to interrupt work with short bouts of exercises or active breaks [43]. This approach has been effective in reducing musculoskeletal symptoms in office workers [44], [45]. However, both these intervention approaches take workers away from their work tasks and have a potential negative impact on productivity. The third, more recent, approach to workplace activity interventions has been to change how productive tasks are performed, such as the use of standing desks [38], [46] and walking or cycling desks [47], [48]. Incorporating some activity, such as standing or walking, into productive work tasks may be more successful at reducing sedentary behaviours as productivity may be minimally impacted [48].

A weakness in some past workplace interventions may have been the lack of a participative approach to changing behaviours. Participative approaches aim to engage workers and develop a sense of ownership and commitment to change by managers/supervisors and workers working as a team to develop and implement health related programmes [49]. Participatory ergonomics practices [50], [51], have successfully been used to address musculoskeletal complaints in industrial [23] and office workplaces [49], [52]–[54] but are yet to be tested for sedentary behaviour interventions.

Past interventions may also have not taken sufficient account of physical and psychosocial features of an organisation that can influence the physical and psychosocical well-being of workers [55], [56]. Organisational features may also impact on the ability of workers to modify work practices in order to change activity and sedentary behaviours. Therefore, organisational characteristics may influence both the sedentary exposure of workers and their response to interventions.

Despite growing evidence indicating the importance of sedentary behaviour in the workplace, to date, there is very limited evidence on the efficacy of workplace interventions to specifically reduce sedentary time. The first aim of this study was to determine if participatory workplace programmes could reduce total sedentary time and sustained sedentary time; increase the frequency of breaks in sedentary time (break rate); and increase the duration of light intensity physical activity and MVPA, on work days and during work hours. Secondly, the study aimed to determine if the intervention effects were consistent across different organisations. The third aim was to determine if a participatory workplace intervention that targeted ‘active’ office work was more effective at reducing sedentary time on work days and during work hours than a participatory workplace intervention targeting non-work activity (traditional physical activity intervention) and an office ergonomics participatory intervention.

## Materials and Methods

### Design

A randomised controlled trial (Australian New Zealand Clinical Trials Registry number: ACTN12612000743864) was conducted with office workers (clerical, data entry and call centre workers) from 3 government organisations in Perth, Western Australia. The protocol for this trial and supporting CONSORT checklist are available as supporting information; see Checklist S1 and Protocol S1. We employed a parallel arms clustered randomised controlled design to compare total sedentary time and sustained sedentary time on work days and during work time following the 12 week intervention period. The trial was not registered prospectively because our study did not focus on health outcomes, but rather on activity. Each organisation formed 3 groups of volunteers based on physical proximity. At Organisation 1 the groups were working on separate floors of the same building. At Organisation 2 the groups were at separate locations on the same floor of the building and at Organisation 3 the groups were in 3 different suburbs. We aimed to have approximately equal numbers in each group.

**Figure 1:**
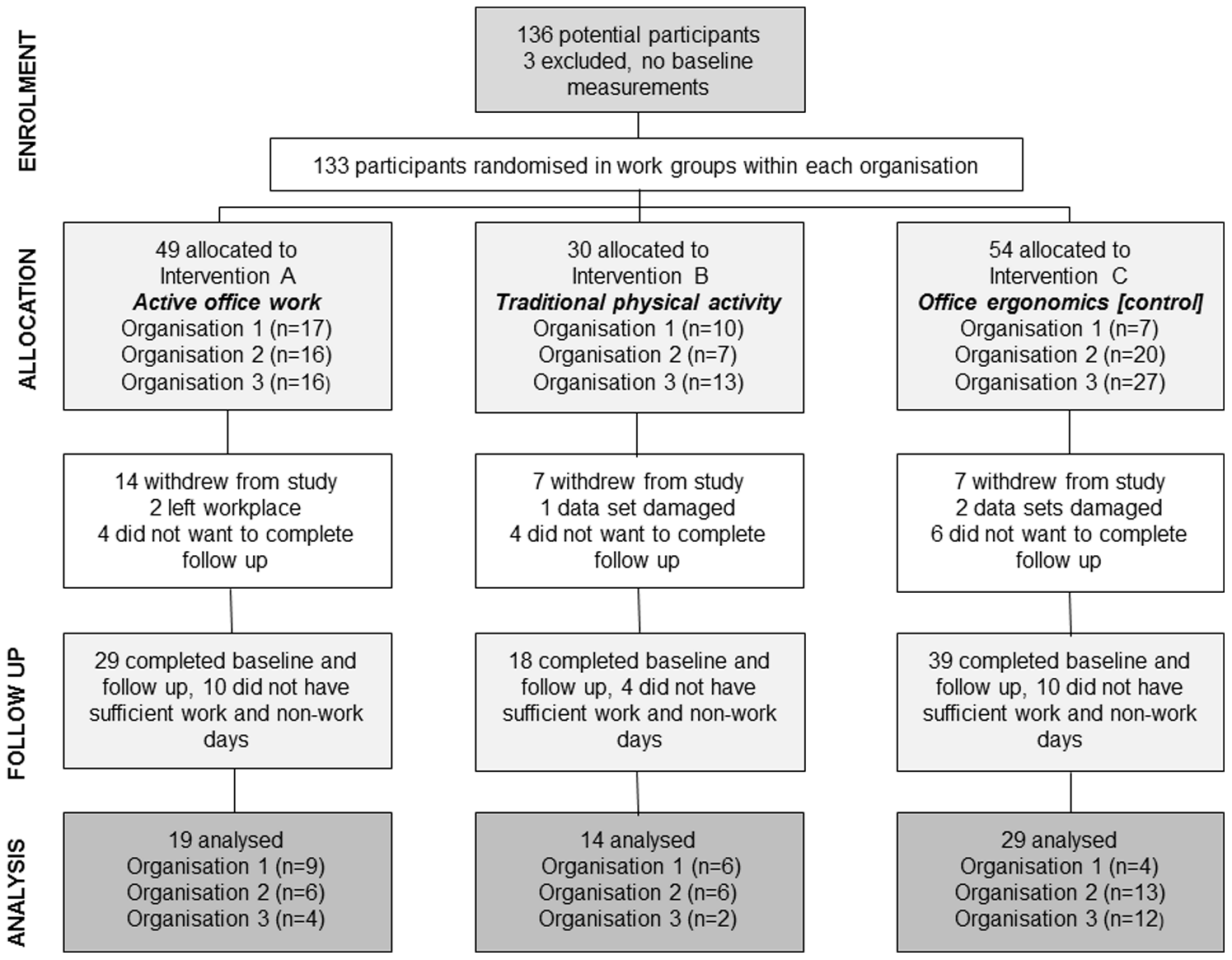
Diagram of the flow of participants through the study.

Within each organisation the groups of physically proximal volunteers were randomly assigned to one of three interventions: A ‘active office work’ intervention, B ‘traditional physical activity’ intervention or C ‘office ergonomics’ intervention (Figure 1). Simple randomisation with a1∶1∶1 allocation ratio was used by drawing a sealed envelope containing the intervention allocation from a hat. This was repeated at each organisation by one of the researchers (SP).

### Participants

Workers participating in office bound duties for 6 or more hours per day and working 4 or more days per week were invited to participate in the study. Participants were only excluded from the study if they were unable to wear an accelerometer due to disability or if they were confined to a wheelchair. Potential participants were recruited at regular monthly staff meetings attended by 20–30 staff. The aim was to recruit 120 participants (40 in each intervention group) to have sufficient power (85%) to detect a 10% change in activity (at alpha = .05) between any 2 intervention groups assuming a standard deviation of the percent change in activity of 15.

### Ethics Statement

All participants provided written informed consent and ethics approval was obtained from the Human Research Ethics Committee, Curtin University (HR20/2007).

### Organisations

The 3 large government organisations had many branches spread across Australia. The recruitment meetings were held at suburban branches that employed between 100–500 people. The nature of the office work and the organisational features varied between the organisations. Organisation 1 was primarily concerned with data processing of large complex files. Workers were able to manage their own time and had flexible working hours and breaks. Organisation 2 was a call centre that handled calls ranging from less than a minute to more complex calls lasting many minutes. Data processing days were scheduled every 3–4 days to provide some job variation. In this organisation, meetings, work breaks and work hours were set by the national office in another city, so that there was very little autonomy or flexibility. Further, productivity, call volume and breaks were monitored and reported on a weekly basis. Organisation 3 was also a data processing workplace where workers were required to process a certain number of documents per day and at times were required to make calls or assist in a call centre. Work hours and breaks were scheduled on site but again these were strictly controlled. Productivity and work compliance were also monitored.

### Interventions

Groups allocated to Intervention A, ‘active office work’, developed interventions aimed at modifying the way office workers completed their tasks with the goal of reducing sedentary time and introducing some light intensity activity while working. Participants in Intervention A had access to a single ‘Active Workstation’ which consisted of an electronically height adjustable desk with integrated treadmill (A7TR78928H, Steelcase, Sydney, Australia; Organisations 1 and 3) or a treadmill plus a stationary cycle ergometer (LF-2850, Exertec Air Bike, Pennsylvania, USA; Organisation 2). It was recommended that the Active Workstation be used for short periods several times a day, starting at 10 minutes and building up to 30 minutes per session. The workstation was equipped with a computer terminal and phone so that normal office duties could be performed. Intervention B, ‘traditional physical activity’, focussed on strategies to promote light to moderate activity in breaks between productive work times and increasing the use of active transport before and after work. Participants in Intervention B were all provided with a pedometer (Yamax Digi-walker SW700, Tokyo, Japan) to use as a motivational tool. Intervention C, ‘office ergonomics’, focussed on computer workstation setup, ‘active’ sitting (moving whilst in the chair) and breaking up computer tasks. Table 1 lists the intervention component details as determined by the intervention groups.

**Table 1:** List of group determined interventions.

Intervention A	Intervention B	Intervention C
***Active office work***	***Traditional physical activity***	***Office ergonomics [control]***
**Active Workstation:** aim for all volunteers tohave 30 minutes daily access	**Pedometer Challenge:** increase walkingduring the work day	“**Active” sitting** – spending some time perching onedge of chair, encouraging movement during sitting
Standing or exercises between calls/documentprocessing	Promote active transport -walk insteadof bus	Taking breaks from sitting
Walk and talk meetings	Walk and talk meetings*	Standing meetings*
Active e-mails – personally delivering informationrather than sending an e-mail*	Short frequent walks during breaks, lunchtime,to and from work*	Use of “piano stool” – reinforcingactive sitting
Increase incidental activity in and around workplace –take longer routes to printer, scanner etc	Increase use of stairs	Use of air cushion

* Common interventions in intervention groups.

### Procedure

Participants from all 3 interventions were asked to attend two structured meetings at their workplace to discuss and develop interventions. A participatory approach to intervention development was used [23] so that workplace interventions could be tailored to the specific needs of the workplace and the employee participants had ownership of the intervention. Prior to the first meeting, baseline body measurements (height and weight) were taken and participants were asked to wear an ActiGraph (GT3X, Pensacola FL) accelerometer for 7 days [57]. The accelerometer was set to record data using a 60 second epoch [58] and attached to an elastic belt to be worn over the right hip [59] for all waking hours. Activities, accelerometer wear time, the reason why the accelerometer was removed (e.g bathing, contact sports), waking hours and work hours (from the time seated at a desk/workstation until leaving the office) were recorded in a simple activity diary.

**Figure 2:**
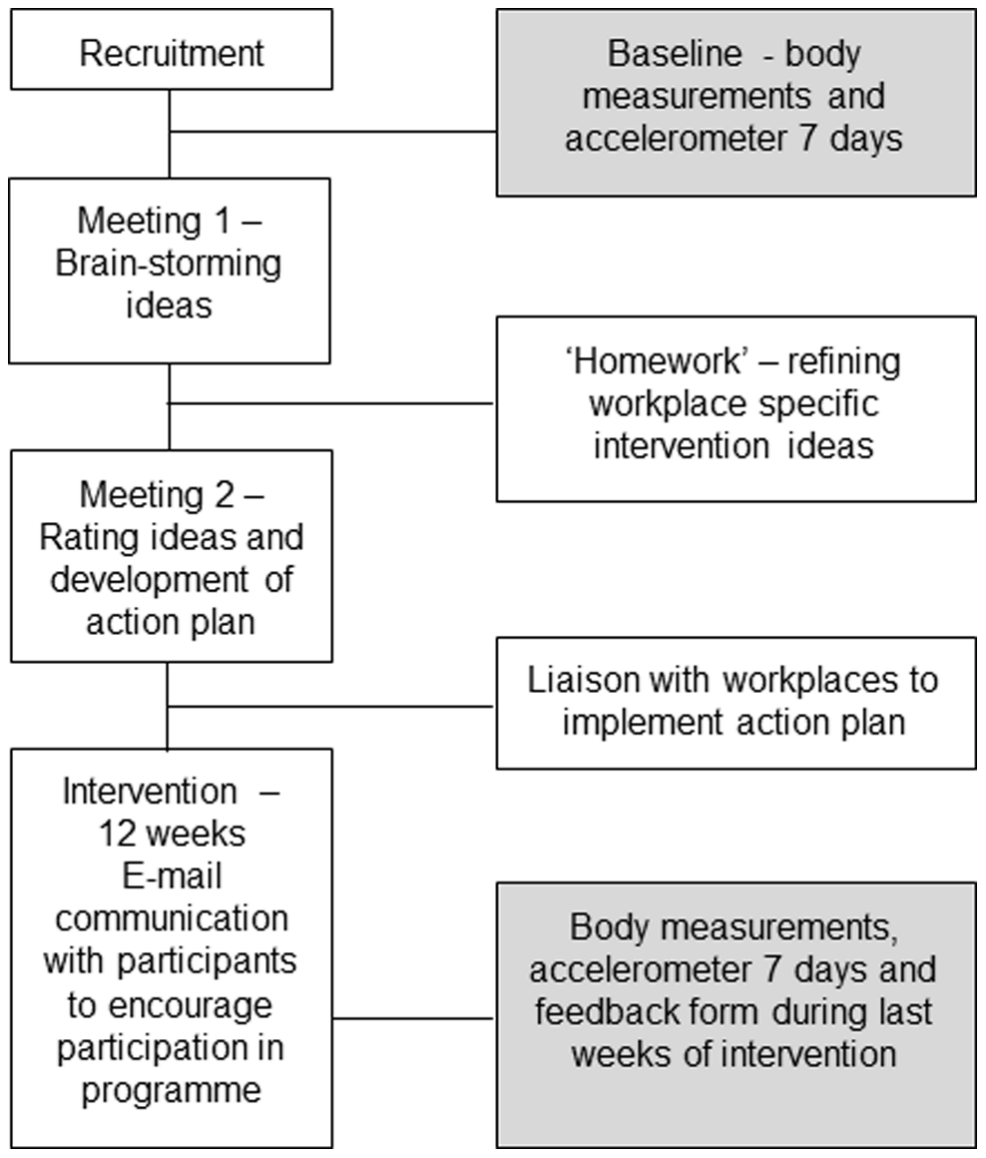
Diagram of the flow of procedures involved in each intervention.

The structured meetings were run by a facilitator (SP). During the first meeting participants ‘brain stormed’ options to promote their specific intervention (active office, physical activity or office ergonomics). Between meetings participants were encouraged to think about specific strategies. At the second meeting, 2–3 weeks following the first meeting, participants shared their ideas and rated the potential strategies in terms of feasibility and effectiveness. At this meeting an action plan was developed and the facilitator communicated with team leaders and management to help implementation. Within 4–6 weeks of the second meeting strategies to be used were in place and the intervention phase was considered to have commenced. Throughout the intervention period, in order to communicate with and motivate participants, tailored emails were sent to each participant by a facilitator (SP) every 2–3 weeks. During the last 2–3 weeks of the intervention, participants had follow up body measurements taken, wore an accelerometer for 7 days and were asked to complete a feedback form to assess participation rate, strengths and barriers for each specific intervention (see Figure 2).

### Outcome Measures

The dual primary outcomes for this study were the total sedentary time and sustained sedentary time on work days and during work time following the intervention period. Secondary outcomes included total light activity time, and frequency of breaks in sedentary time during work periods. Activity time and breaks were based on accelerometer data. The researcher with primary responsibility for collection and analysis of accelerometer data (SP) had conducted the interventions and was not blinded to group allocation. The ActiGraph data were downloaded using the *ActiLife 5* software (ActiGraph, Pensacola FL) and then activity count data were processed using a custom program (LabVIEW 8.6.1 National Instruments, Texas, USA). The program enabled detailed simultaneous analysis of the pattern of activity intensity and duration to be studied using Exposure Variance Analysis [60], [61]. Activity intensity categories of sedentary, light, moderate and vigorous were determined from the Freedson et al. [62] and Matthews et al [63] counts per minute cut points (sedentary<100 counts, light 100-<1951 counts, moderate 1951-<5275 counts and vigorous >5275). Duration was characterised as bouts within the same intensity lasting 0-<5 mins, 5-<10 mins, 10-<30 mins, 30- <60 mins and 60+ mins to match other research and recommendations [64]–[66]. Non-wear time during waking hours was determined by firstly examining the activity diary and then during the accelerometer processing, where periods with greater than 120 minutes of consecutive zeros were considered non-wear time. A break in sedentary time was defined as accelerometer counts above 100 counts/min for greater than one minute during sedentary time [2]. Minimum wear time was set at 500 minutes/day [67], [68] and at least 3 work days and 1 non-work day was required for inclusion [69], [70].

### Statistical Analysis

Independent t-tests or chi squared tests evaluated differences in participant characteristics and baseline activity levels between participants that completed the study with sufficient data and those who did not. One way ANOVA or chi squared tests compared baseline differences between organisations and between intervention groups. For the first aim, repeated measures t tests were used to test overall effect of any intervention for all participants. For aims two and three, linear regression models (ANCOVA) for each outcome were used to estimate the magnitude and corresponding 95% confidence intervals of intervention effects, with the post-intervention measures as the dependent variable, the 3-level categorical variables ‘organisation’ and ‘intervention’ as independent variables and the corresponding baseline measure as a covariate. This allowed intervention effects to be adjusted for differences between organisations. Robust standard errors were specified due to potential non-independence of observations for individuals within organisations. No adjustment for multiple testing was made to balance Type 1 and Type 2 errors. Analysis was conducted using the intention to treat assumption that participants allocated to a particular intervention received that intervention. Activity analyses were calculated using percentage of wear time for each time period, with all analyses performed using PASW Statistics 18 or Stata/IC 12.1 for Windows (StataCorp LP, TX USA; critical alpha level of 0.05).

## Results

### Participant Characteristics at Baseline

133 volunteers (82% female) aged between 20 and 65 years (mean ± SD; 41.4±10.9 years) with a BMI of 28.4±6.4 kg/m^2^ completed the baseline measurements. Data were collected in 2010–2011 and analysed in 2012. The trial was ended due to the lack of further organisations willing to participate within the two year data collection period. 28 participants withdrew from the study during or after the workplace meetings and did not take part in the intervention. A further 14 did not want to complete the follow up analysis (body measurements and accelerometry), 3 sets of accelerometer data were lost due to equipment failure and 2 participants left the workplaces. 24 data sets had insufficient work or non-work days to be included in the analyses. No adverse outcomes were reported for any participants. As shown in Figure 1, 62 participants had complete data sets and were included in analyses (81% female; 43.5±6.4 years and BMI 28.0±6.4 kg/m^2^). Those analysed did not differ from those that were not analysed, in BMI, time in baseline activity levels on work days and during work hours. However, they were significantly older and wore their accelerometer for less time on work days (Table 2).

**Table 2:** Comparison of participant characteristics and activity levels at baseline between participants that were analysed and those not included in analysis.

Variable	Analysed Participants(n = 62)	Non-analysed Participants(n = 71)	p for groupcomparison^1^	Difference(95% CI)
Age (mean years; [SD])	43.5 [6.4]	39.3 [11.8]	0.03	−4.2 (−7.88, −0.43)
Gender (n (%) female)	50 (80.6)	59 (83.1)	0.71	−2.5% (−11, 16)
BMI (mean kg/m^2^; [SD])	28.0 [6.4]	28.7 [6.4]	0.55	0.7 (−1.55, 2.91)
Wear time work day(mean mins; [SD])	921.9 [83.8]	862.5 [87.3]	0<0.001	−59.4 (−88.8, −29.9)
Wear time work hours(mean mins; [SD])	501.8 [65.3]	495.7 [42.8]	0.52	−6.1(−24.82, 12.63)

1 Independent t-tests for age, BMI and wear time; chi squared for gender.

### Intervention Effect on Sedentary Time, Sustained Sedentary Time, Light Activity, MVPA and Break Rate for All Participants on Work Days and during Work Hours

Sedentary time, sustained sedentary time, break rate, light activity and MVPA before and after the intervention period are presented in Table 3. Overall, there was a significant reduction in the percentage of sedentary time on work days (−1.6%) and during work hours (−1.7%). It was estimated that the percentage of sustained sedentary time decreased by −2.1% on work days and by −3.2% during work hours, though these changes were not statistically significant (Table 3). The reduction in sedentary time of 1.7% during work hours is equivalent to 8 less sedentary minutes during work hours. There was also a significant increase in the break rate (number of breaks/sedentary hour) for all participants on work days and during work hours (Table 3).

**Table 3:** Sedentary time, light activity, MVPA, sustained sedentary time (bouts>30 mins) and break rate (breaks/sedentary hour) for all participants before and after intervention.

Outcome measures	Pre-intervention	Post intervention	Mean	95% CI	*P* 1
	(% wear time ± SD)	(% wear time ± SD)	Change		
***Sedentary time***					
Work days	72.85±7.06	71.25±7.27	−1.60	−0.48, −2.72	0.006
Work hours	78.29±8.41	76.6±8.6	−1.71	−0.37, −3.06	0.014
***Sustained Sedentary time***					
Work days	24.37±12.73	22.29±13.16	2.08	−0.47, 4.62	0.108
Work hours	28.98±19.34	25.74±18.66	3.24	−0.63, 7.11	0.099
***Break rate***					
Work days	7.81±2.45	8.45±2.86	0.64	1.08, 0.20	0.005
Work hours	6.95±3.20	7.67±3.41	0.72	1.29, 0.15	0.015
***Light time***					
Work days	23.85±6.37	24.81±6.48	0.97	2.11, −0.18	0.098
Work hours	19.14±7.75	20.63±7.86	1.49	2.87, 0.10	0.036
***MVPA***					
Work days	3.29±1.83	3.93±2.34	0.64	1.13, 0.14	0.012
Work hours	2.57±1.83	2.79±1.83	0.22	0.69, −0.24	0.334

1 Paired t-test between pre- and post-intervention values.

It was estimated that the percentage of light activity on work days increased by 1.0%, but this was not statistically significant. However, the estimated increase during work hours of 1.5% was statistically significant. Similarly, the estimated 0.6% increase in MVPA on work days was statistically significant but the estimated increase of 0.2% in MVPA during work hours was not significant (Table 3). The 1.5% increase in light activity during work hours is equivalent to 7 more light intensity minutes during work hours.

### Intervention Effects Across the Organisations

There were significant differences between organisations at baseline for sedentary time during work hours (F_2,59_ = 3.80, p = 0.028), MVPA during work hours (F_2,59_ = 5.02, p = 0.010) and for break rate during work hours (F_2,59_ = 3.18, p = 0.049).

After adjusting for baseline measures and type of intervention, pre- to post-intervention changes in sedentary time, sustained sedentary time, light activity, MVPA and break rate during work hours differed by organisation with Organisation 1 responding most to interventions and Organisation 3 responding least (Table 4). For example, it was estimated that the reduction in percentage of sedentary time during work hours (adjusted for type of intervention and baseline) was −4.1, −1.3 and 0.1 for Organisations 1, 2 and 3 respectively; which equated to an adjusted difference of 2.8 (95%CI: −0.8, 6.4, p = 0.120) between Organisations 1 and 2, and 4.2 (95%CI: 0.6, 7.7, p = 0.021) between Organisations 1 and 3.

**Table 4:** Results of multivariable linear regression analysis for sedentary, sustained sedentary, light, moderate/vigorous physical activity time and break rate during work hours.

Outcome measures	Adjusted Pre- to post-intervention change^1^(95% CI)	GroupDifferences in change(REF - group)(*β*(95% CI))	*P*
**Sedentary time work hours (% wear time)**			
**Intervention**			0.325^2^
Active Office - A	−3.09	REF	
	(−5.82, −0.35)		
Office Ergonomics - C	−1.37	−1.72	0.289
	(−2.86, −0.13)	(−4.94,1.50)	
Physical Activity - B	−0.57	−2.52	0.248
	(−3.54,2.40)	(−6.84,1.80)	
**Organisation**			0.043^2^
Organisation 1	−4.07	REF	
	(−6.70, −1.43)		
Organisation 2	−1.26	2.80	0.120
	(−3.32, −0.79)	(−0.75,6.36)	
Organisation 3	0.14	4.21	0.021
	(−1.71,2.00)	(0.66,7.76)	
**Sustained sedentary time (sedentary bouts>30 mins) work hours (% wear time)**			
**Intervention**			0.485^2^
Active Office - A	−2.87	REF	
	(−9.23,3.49)		
Office Ergonomics - C	−5.60	2.73	0.495
	(−10.29, −0.91)	(−5.22,0.69)	
Physical Activity - B	1.17	−4.04	0.486
	(−7.24,9.58)	(−15.55,7.48)	
**Organisation**			0.046^2^
Organisation 1	−8.64	REF	
	(−14.65, −2.64)		
Organisation 2	−3.84	4.81	0.212
	(−9.03,1.35)	(−2.81,12.43)	
Organisation 3	3.31	11.95	0.014
	(−3.49,10.11)	(2.55,21.35)	
**Light activity work hours (% wear time)**			
**Intervention**			0.616^2^
Active Office - A	2.53	REF	
	(−0.42,5.49)		
Office Ergonomics - C	1.38	1.16	0.497
	(−0.06,2.81)	(−2.23,4.54)	
Physical Activity - B	0.29	2.24	0.328
	(−2.75,3.33)	(−2.31,6.80)	
**Organisation**			0.124^2^
Organisation 1	3.57	REF	
	(0.84,6.29)		
Organisation 2	1.07	−2.50	0.189
	(−1.12,3.27)	(−6.26,1.26)	
Organisation 3	−0.14	−3.71	0.044
	(−1.95,1.68)	(−7.30, −0.11)	
**Moderate-vigorous activity work hours (% wear time)**			
**Intervention**			0.136^2^
Active Office - A	0.97	REF	
	(0.06,1.88)		
Office Ergonomics - C	−0.17	1.15	0.047
	(−0.66,0.31)	(0.02,2.27)	
Physical Activity - B	0.04	0.93	0.189
	(−0.89,0.98)	(−0.47,2.33)	
**Organisation**			0.032^2^
Organisation 1	0.69	REF	
	(−0.14,1.51)		
Organisation 2	0.42	−0.27	0.630
	(−0.28,1.11)	(−1.39,0.85)	
Organisation 3	−0.53^3^	−1.21	0.024
	(−1.03, −0.02)	(−2.26, −0.17)	
**Break Rate (breaks/sedentary hour)**			
**Intervention**			0.382^2^
Active Office - A	0.85	REF	
	(−0.33,2.02)		
Office Ergonomics - C	0.97	−0.12	0.871
	(0.24,1.69)	(−1.57,1.33)	
Physical Activity - B	0.02	0.83	0.355
	(−1.14,1.18)	(−0.95,2.61)	
**Organisation**			0.058^2^
Organisation 1	1.75	REF	
	(0.72,2.78)		
Organisation 2	0.45	−1.30	0.094
	(−0.51,1.42)	(−2.82,0.22)	
Organisation 3	−0.01	−1.76	0.018
	(−0.86,0.84)	(−3.20, −0.31)	

1 Intervention estimates adjusted for baseline and organisation, Organisation estimates adjusted for baseline and intervention.

2 Omnibus p-value for overall group difference.

3 Also significantly different to Organisation 2 by −0.94 (95%CI: −1.84, −0.04, p = 0.040).

### Effect of the Different Interventions on Sedentary Time, sustained sedentary Time, Light Activity, MVPA and Break Rate during Work Hours

At baseline there were no significant differences in BMI (F_2,59_ = 0.22, p = 0.803), age (F_2,59_ = 0.03, p = 0.969), gender (χ^2^ = 4.25, p = 0.119) or wear time during work hours (F_2,59_ = 2.71, p = 0.075) between the three intervention groups. There were significant differences between intervention groups at baseline in sedentary time (F_2,59_ = 4.21, p = 0.020), sustained sedentary time (F_2,59_ = 4.02, p = 0.023) and light intensity activity (F_2,59_ = 3.41, p = 0.040) during work hours. In addition to baseline differences between interventions, there was some imbalance in intervention allocation across organisations (see Figure 1). Therefore, linear regression analyses to assess differences in the effect of type of intervention were adjusted for organisation in addition to the standard procedure of adjusting for baseline measures.

Whilst Intervention A appeared to be associated with greater change, after adjustment for baseline measures and organisation no one intervention was more effective at changing the amount of sedentary time, sustained sedentary time, light activity, MVPA or break rate during work hours. For example it was estimated that Interventions A, B and C resulted in a reduction in the percentage of sedentary time during work hours (adjusted for organisation and baseline) of −3.1, −0.6 and −1.4 for Interventions A, B and C respectively; however the adjusted differences of −1.7 between A and C (95% CI: −4.9, 1.5, p = 0.289) and −2.5 between A and B (95% CI: −6.8, 1.8, p = 0.248) were not significant (Table 4).

## Discussion

This unique study examined three workplace interventions to reduce sedentary time and sustained sedentary time of office workers using a participatory approach to intervention development and implementation. Overall the interventions resulted in a significant reduction in sedentary time and a concurrent increase in light intensity activity during work hours. There was also an increased break rate (breaks/sedentary hour) during work hours. Intervention effects were greatest in Organisation 1. None of the 3 interventions (active office work, traditional physical activity and office ergonomics) was clearly more effective at improving occupational sedentary behaviour.

Whilst the interventions resulted in improved occupational sedentary behaviour, the changes were small, in the order of 1–2% during work hours. Currently, there is uncertainty as to what amount of sedentary time will adversely affect health, that is, what is the minimally clinically important difference. In large population studies, Healy et al [8] found that in the most sedentary sub-group, for every one hour/day increase in sedentary time, waist circumference increased by 1.4 cm. Further, Camhi [71] found that for increases in light activity of 30 minutes there were lowered odds of between 33–54% for reduced blood cholesterol and waist circumference. In the present study, there was an average reduction in sedentary time of 8 minutes and increases in light activity of 7 minutes during work hours. Whether changes of this magnitude are sufficient to change health risk is not known yet. Recent studies have demonstrated that 28 minutes of light activity in 2 minute bouts resulted in positive effects on glucose metabolism [10] indicating that small changes such as those found in the present study have the potential to positively impact on the health of sedentary workers.

Organisations 2 and 3 involved call centre and data processing work and showed the least change in sedentary time, sustained sedentary time and break rate during work hours. In these organisations, productivity and compliance measures were monitored regularly and employees had the least amount of work flexibility and control with little opportunity to vary their work tasks or even when to take coffee and meal breaks. Therefore, in order to create meaningful and sustainable changes in sedentary time, in arguably the most challenging and sedentary group of office workers, sedentary work practices needed to change. Workplace practices within the organisations that participated in the study were regimented so that varying office tasks to incorporate incidental activity, such as longer walks to the printer were difficult to implement. Feedback from the participants indicated that these interventions were not fully supported by the management/team leaders within the organisations. Even though management and participants were aware of the intervention options, changing the organisational culture in these workplaces had limited success and such change may require stronger external support such as guidelines. Emerging sedentary guidelines [5], [72] are recommending similar behaviour changes to the ergonomic guidelines to prevent musculoskeletal pain in computer work developed in the late 20^th^ century, such as reduced screen time and increased variation in work tasks [73]. Implementation of sedentary guidelines may be particularly important in this vulnerable group of office workers in order to effect change in occupation sedentary behaviour.

There are number of potential reasons for why there did not appear to be one intervention that was clearly superior to the others in terms of reduced sedentary time on work days and during work hours. Participants from all intervention groups took part in workplace meetings to develop workplace specific interventions as part of the participatory approach. As a result of the consulting process, there were overlapping intervention ideas so that some of the interventions strategies implemented were common across the intervention groups. Further, the active office and physical activity interventions were very similar for most participants as only a few participants used the Active Workstation and then usually only to a limited extent. Feedback from the participants indicated barriers to use of the Active Workstation included the time taken to log on and off their regular computer, an unfamiliar workstation and fear of perceived loss of productive work time. Replacing a standard desk with a ‘treadmill desk’ [74] or incorporating a standing workstation into standard desks [38] has recently been more successful in changing occupational sedentary activity than providing standing ‘hot’ desks [46] or an isolated Active Workstation such as the one used in this study. The success of each of the interventions may also be indicative of the participatory approach ensuring a match between the work group and the variety of strategies available to encourage occupational incidental activity and reduced occupational sedentary time.

A strength of this study was that it was a randomised controlled study examining a variety of interventions to reduce sedentary time and sustained sedentary time. Further, the use of a participatory approach resulted in interventions that were workplace specific. Previous studies have used convenience samples of university employees [38], [39], [75] whereas this study attempted to modify the work practices of office workers in typical situations where there was very little flexibility in the work environment, and thus had high external validity.

Limitations in the study include the modest number of participants that completed the study. There were only half the number of participants that were planned based on the initial power calculations. Whilst this provided sufficient power to detect the 1.7% difference in sedentary time during work hours across all participants (first aim), it did not provide sufficient power to detect small differences such as the 2.5% observed in this sample (third aim). Also, the number of intervention strategies implemented in each intervention group and the similarities between the interventions as implemented and the imbalance between the group sizes within the organisations meant that the efficacy of particular intervention components could not be determined. The sample size and differences in group sizes between organisations also restricted the use of mixed effect models or generalised estimating equations and the lack of alpha level adjustment for primary hypothesis testing may not have balanced Type 1 and Type 2 errors optimally.

This study demonstrated that consultation with employees, managers and team leaders using a participatory approach could achieve tailored workplace interventions that resulted in modest changes to sedentary behaviour in a group of particularly vulnerable office workers. Future research should try to determine more effective interventions, how to match interventions to organisational features, minimally clinically important differences for sedentary behaviour in general, and dose-response relationships between occupational sedentary behaviour and various health outcomes. Revising the workplace guidelines developed in the 20^th^ century to reduce musculoskeletal disorders should also be extended to incorporate knowledge about the importance of sedentary behaviour and light activity in the reduction of cardiometabolic disorders.

## Conclusion

Participatory workplace activity programmes can reduce sedentary time during work hours. The reduction in sedentary time was associated with an increase in light intensity activity and in the number of breaks in sedentary time during work hours. While the changes were small, this study highlighted the potential for making modifications to office work and exploring novel ways, such as the use of an Active Workstation, to reduce occupational sedentary behaviour.

## Supporting Information

Checklist S1 (DOC)

Protocol S1 (PDF)
