# Supplementary material for: Participatory Workplace Interventions Can Reduce Sedentary Time for Office Workers—A Randomised Controlled Trial
Source: PLoS One. 2013 Nov 12;8(11):e78957. doi: 10.1371/journal.pone.0078957 (PMC3827087; doi:10.1371/journal.pone.0078957)
Supplement: Protocol S1 — (PDF) [file pone.0078957.s002.pdf]

ANZCTR
Australian New Zealand Clinical Trials Registry

Questions in **bold** text are mandatory. **(*)**

**Request Number:**	362738
**Current Page:**	Review

Trial from ANZCTR

**Trial ID**	ACTRN12612000743864
**Trial Status:**	Registered
**Date Submitted:**	9/07/2012
**Date Registered:**	12/07/2012
	**Retrospectively registered**

Page 1

**Public title**	Can a participatory workplace intervention improve sedentary behaviour and physical activity in office workers?
**Study title in 'Participant-Intervention-Comparator- Outcome (PICO)' format**	In office workers, is a participatory workplace intervention focussed on work tasks more effective than participatory workplace interventions focussed on discretionary time or workstation ergonomics in reducing sustained sedentary time and increasing physical activity?
**Secondary ID [1]**	Nil
UTN	
Trial acronym	

Page 2

**Health condition(s) or problem(s) studied:**	
Sedentary behaviour	
**Condition category:**	**Condition code:**
Public Health	Health promotion/education

Page 3

**Descriptions of intervention(s) / exposure**	All participants will be involved in one of three workplace based interventions of 12 weeks duration. Prior to the intervention participants will attend 2 structured workplace meetings each of 45-60 minutes duration with the members of their intervention group. At the first meeting, the participants will be presented with information on the important health implications of office work and discuss potential changes they could make around the focus of their intervention. At the second meeting, approximately 2 weeks after the first meeting, participants will discuss ideas for change, develop the strategies and rate their ease of implementation and likely effect, again with the assistance of a facilitator. Work groups will then be given 4-6 weeks to organise the implementation of their chosen strategies including getting organisational approval. At this point the 12 week intervention period will be deemed to have begun. There will be 2 intervention groups and an 'active' control group which will develop interventions in line with their focus as listed below:<br><br>(1) Work task modification focus to reduce sedentary behaviour and increase physical activity. For example, provision of an ‘active workstation’ which is an electronic height adjustable desk that is connected to a treadmill or exercise bike enabling participants to ‘walk and work’. Other interventions may include changing default printers to a more distant location or walking to communicate with colleagues rather than emailing - to encourage incidental activity during work tasks in the workplace.<br><br>(2) Discretionary time focus to reduce sedentary behaviour and increase physical activity. For example walking during work breaks (lunch/coffee) and before and after work. Participants in this group may be provided with a pedometer for use in a ‘steps challenge’. Other interventions may include promotion of active transport, groups exercise classes or lunchtime walking groups.<br><br>(3) 'Active' control focussed on office workstation ergonomics review and modification. For example, revision of workstation set up and the introduction to ‘active sitting’, a sitting technique that encourages trunk muscle activity while sitting. Participants may be provided with an ‘air cushion’ that facilitates active sitting.
**Intervention Code:**	Prevention
**Intervention Code:**	Behaviour

**Comparator / control treatment**	'Active' control focussed on office workstation ergonomics review and modification. For example, revision of workstation set up and the introduction to 'active sitting', a sitting technique that encourages trunk muscle activity while sitting. Participants may be provided with an 'air cushion' that facilitates active sitting.
**Control group**	Active

Page 4

**Primary Outcome:**	Total sedentary time assessed with Actigraph triaxial accelerometer worn at waist level for 7 days. Accelerometry data assesses the duration and intensity of physical activity on work days and weekend days and during work time and outside of work time.
Timepoint:	Before intervention, during last week of 12 week intervention period and 3 months post intervention period
**Primary Outcome:**	Sustained sedentary time (periods of uninterupted sedentary time of greater than 30 minutes) assessed with Actigraph triaxial accelerometer worn at waist level for 7 days. Accelerometry data assesses the duration and intensity of physical activityon work days and weekend days and during work time and outside of work time.
Timepoint:	Before intervention, during last week of 12 week intervention period and 3 months post intervention period
**Secondary Outcome:**	Total light activity time assessed with Actigraph triaxial accelerometer worn at waist level for 7 days. Accelerometry data assesses the duration and intensity of physical activity on work days and weekend days and during work time and outside of work time.
Timepoint:	Before intervention, during last week of 12 week intervention period and 3 months post intervention period
**Secondary Outcome:**	Frequency of breaks in sedentary time assessed with Actigraph triaxial accelerometer worn at waist level for 7 days. Accelerometry data assesses the duration and intensity of physical activity on work days and weekend days and during work time and outside of work time.
Timepoint:	Before intervention, during last week of 12 week intervention period and 3 months post intervention period
**Secondary Outcome:**	Self reported sitting time assessed by International Physical Activity Questionnaire (IPAQ) (Craig et al, 2003)
Timepoint:	Before intervention, during last week of 12 week intervention period and 3 months post intervention period
**Secondary Outcome:**	Self reported physical activity time assessed by International Physical Activity Questionnaire (IPAQ) (Craig et al, 2003)
Timepoint:	Before intervention, during last week of 12 week intervention period and 3 months post intervention period
**Secondary Outcome:**	Self reported musculoskeletal pain assessed by modified Nordic musculoskeletal pain questionnaire (Kuorinka et al, 1987)
Timepoint:	Before intervention, during last week of 12 week intervention period and 3 months post intervention period
**Secondary Outcome:**	Self reported job satisfaction modified Job Satisfaction Survey (Warr, 1990)
Timepoint:	Before intervention, during last week of 12 week intervention period and 3 months post intervention period
**Secondary Outcome:**	Readiness for physical activity self assessed by readiness for physical activity questionnaire which categorises subjects into stages of change categories (Precontemplation, Contemplation, Action and Maintenance) (Marcus et al, 1992)
Timepoint:	Before intervention, during last week of 12 week intervention period and 3 months post intervention period
**Secondary Outcome:**	Self reported health and productivity modified World Health Organisation Health and Work Performance Questionnaire (HPQ) (Kessler et al, 2003)
Timepoint:	Before intervention, during last week of 12 week intervention period and 3 months post intervention period
**Secondary Outcome:**	Qualitative feedback about the success and barriers to the intervention - tailored feedback form
Timepoint:	Last week of intervention period and 3 months post intervention

Page 5

**Key inclusion criteria**	Office based workers working 4 or more days per week, for 6 or more hours each work day
**Minimum age**	18 Years
**Maximum age**	No limit
**Gender**	Both males and females
**Healthy volunteers?**	Yes
**Key exclusion criteria**	Physical or psychological impairments that would interfere with their ability to participate in the trial, for example being wheelchair bound

Page 6

**Study type**	Interventional
**Purpose of the study**	Prevention

**Allocation to intervention**	Randomised controlled trial
Describe the procedure for enrolling a subject and allocating the treatment (allocation concealment procedures)	Participants in each organisation will be divided into 3 groups based on the physical location of the teams that they work in. Each group within the workplace will be randomly allocated (sealed envelope) into one of the intervention groups or the control group
Describe the methods used to generate the sequence in which subjects will be randomised (sequence generation)	Randomisation will be to one of the 3 interventions (2 focussed on physical activity, 1 'active' control focussed on workstation ergonomics). Simple randomisation sequence will therefore be used as only only 3 options are required. Sealed envelopes will be drawn from a box at each organisation.
Masking / blinding	Open (masking not used)
Who is / are masked / blinded (choose all that apply)	
Assignment	Parallel
Other design features	Clustered randomised and controlled trial - where three physically separated groups of workers in each participating organisation will be randomised to the intervention and control groups.
Type of endpoint (s)	Efficacy
Statistical Methods/Analysis	

Page 7

Phase	Not Applicable
**Anticipated date of first participant enrolment**	1/02/2010
Date of first participant enrolment	
Anticipated date last participant recruited/enrolled	
Actual date last participant recruited/enrolled	
**Target sample size**	120
**Recruitment status**	Closed: follow-up complete

Recruitment in Australia

**Recruitment state(s)**	

Recruitment outside Australia

Page 8

**Funding Source:**	University
**Name:**	Curtin University
**Address:**	GPO Box U1987<br>Perth<br>WA 6845
**Country:**	Australia
**Primary Sponsor**	University
**Name:**	Curtin University
**Address:**	GPO Box U1987<br>Perth<br>WA 6845
**Country:**	Australia
**Secondary Sponsor:**	None
**Name:**	
**Address:**	
**Country:**	
Other Collaborator:	University
Name:	The University of Queensland
Address:	St Lucia<br>Brisbane<br>Qld 4072

Country:	Australia

Page 9

**Has the study received approval from at least one Ethics Committee?**	Yes
Ethics Committee name:	Curtin University Human Research Ethics Committee
Address:	Curtin University<br>GPO Box U1987<br>Perth Wa 6845
Country:	Australia
Approval Date:	01/06/2007
Submitted Date:	
HREC:	HR20/2007
Brief summary	Physical inactivity is one of the major causes of many chronic preventable diseases such as obesity, diabetes and cardiovascular disease. There is a growing understanding that low energy activity or sedentary behaviour and breaks in sedentary time are also independently linked to cardiometabolic risk factors. Furthermore, in the ‟technological” age, occupational physical activity has reduced. Currently, it is estimated that office workers spend approximately 75% of their working hours sedentary (sitting) and are therefore at risk of many of the adverse health consequences associated with prolonged sedentary behaviour. Workplace activity intervention programmes have focussed on increasing moderate/vigorous physical activity typically by encouraging walking before and after work or during breaks. To date, there is no research that has examined the effect of modifying office work to incorporate short bouts of light intensity physical activity and encouraging breaks in sedentary behaviour.<br>Aims<br>1) To assess if a participatory work-based programme to encourage incidental and light intensity physical activity can reduce sedentary time of office workers<br>2) To assess if a participatory work task focussed intervention to promote reduced sustained sedentary time and increased incidental activity is more effective at reducing sedentary time than interventions focussed on physical activity in discretionary time or an office workstation ergonomics intervention<br>3) To assess if changes that result from a participatory work-based intervention programme are sustained 3 months following the intervention<br>4) To determine if there is a relationship between participatory workplace health programmes and musculoskeletal pain and job satisfaction
Trial website	
Trial related presentations / publications	
Public Notes	

Page 10

Principal Investigator

Title:	
Name:	
Address:	
Country:	
Tel:	
Fax:	
Email:	

Contact person for public queries

Title:	
Name:	Professor Leon Straker
Address:	School of Physiotherapy Curtin University of Technology GPO Box U 1987 Perth WA 6845
Country:	Australia
Tel:	+61 8 9266 3634
Fax:	+61 8 9266 3699
Email:	L.Straker@curtin.edu.au

Contact person for scientific queries

Title:	
Name:	Professor Leon Straker
Address:	School of Physiotherapy Curtin University of Technology GPO Box U 1987 Perth WA 6845
Country:	Australia

Tel:	+61 8 9266 3634
Fax:	+61 8 9266 3699
Email:	L.Straker@curtin.edu.au

Contact person responsible for updating information

Title:	
Name:	Professor Leon Straker
Address:	School of Physiotherapy Curtin University of Technology GPO Box U 1987 Perth WA 6845
Country:	Australia
Tel:	+61 8 9266 3634
Fax:	+61 8 9266 3699
Email:	L.Straker@curtin.edu.au
